# Exogenous Nitric Oxide-Induced Postharvest Gray Spot Disease Resistance in Loquat Fruit and Its Possible Mechanism of Action

**DOI:** 10.3390/ijms24054369

**Published:** 2023-02-22

**Authors:** Yanfang Ren, Tengyu Yan, Chunmei Hu, Dong Liu, Junyu He

**Affiliations:** 1 School of Environmental Science and Engineering, Changzhou University, Changzhou 213164, China; 2 College of Agriculture, Guizhou University, Guiyang 550025, China

**Keywords:** *Eriobotrya japonica* L., gray spot disease, nitric oxide, induced resistance, antioxidant enzymes, cell wall metabolism

## Abstract

The effectiveness of nitric oxide (NO) for control of grey spot rot cause by *Pestalotiopsis eriobotryfolia* in harvested loquat fruit and its probable mechanisms have been investigated. The results showed that NO donor sodium nitroprusside (SNP) did not evidently inhibit mycelial growth and spore germination of *P. eriobotryfolia*, but resulted in a low disease incidence and small lesion diameter. SNP resulted in a higher hydrogen peroxide (H_2_O_2_) level in the early stage after inoculation and a lower H_2_O_2_ level in the latter period by regulating the activities of superoxide dismutase, ascorbate peroxidase and catalase. At the same time, SNP enhanced the activities of chitinase, β-1,3-glucanase, phenylalanine ammonialyase, polyphenoloxidase, and total phenolic content in loquat fruit. However, SNP treatment inhibited the activities of cell wall-modifying enzymes and the modification of cell wall components. Our results suggested that NO treatment might have potential in reducing grey spot rot of postharvest loquat fruit.

## 1. Introduction

Loquat (*Eriobotrya japonica* L.) fruit, an important subtropical fruit, is widely cultivated in more than 30 countries [1], being highly appreciated for its mild taste as well as its nutritional and medicinal properties [2]. Due to its thin peel, tender and juicy pulp, as well as the fact that it matures in the rainy and warm seasons, loquat fruit is prone to mechanical damage and decay, resulting in a significant economic loss and short postharvest storage period [3,4,5]. Therefore, extending its shelf life by preserving fruit quality is a crucial objective for postharvest loquat. Traditionally, synthetic fungicide has been widely applied for preventing postharvest decay of loquat fruit [6]. Nevertheless, continuous and exclusive use of fungicides has resulted in the resistance to fungicides and potential harmful effects on human health and the environment [7]. Thus, safe and eco-friendly approaches for controlling postharvest disease in loquat fruit instead of chemical fungicides are required [4,5].

The induction of fruit resistance against pathogenic infection with biological and chemical elicitors has become a promising approach for the control of postharvest disease over recent years [8]. Nitric oxide (NO) is a natural metabolite present in the plant kingdom and plays diverse signal functions, including trigging defense responses to abiotic and biotic stresses in plants [9,10,11]. It was reported that during plant-pathogen interaction, NO levels increased sharply along with enhanced resistance against disease in plants [12]. Recently, influences of NO application or its exogenous donor sodium nitroprusside (SNP) in disease resistance of various fruits have received much attention. Previous studies showed that NO effectively enhanced the resistance of pitaya, citrus, and apple fruit to anthracnose [12,13,14] and tomato fruit to gray mold rot [15] while reducing the disease incidence of kiwifruit [11]. Exogenous NO enhanced the resistance of harvested peach fruit against *Monilinia fructicola* and brown rot disease [16]. NO activated phenylpropanoid metabolism of muskmelon against *Trichothecium roseum* [5]. These findings suggested that NO might be promising as a natural fungicide to partially substitute for the utilization of synthetic fungicides in fruit [17].

The mechanisms of disease resistance by NO or SNP in postharvest fruit included in oxidative burst, the production of antimicrobial phytoalexins, the induction of antioxidant activities, the synthesis of pathogenesis-related (PR) proteins, etc. [11,18]. In addition, some tests in vitro showed that NO did not affect the mycelial growth and spore germination of fungi but significantly reduced disease incidence and lesion areas in fruit [15,19,20]. These studies suggested that the enhanced resistance of fruit to the fungal pathogen caused by NO was indirect. However, some reports indicated that there was a direct link between NO and plant pathogens. Wang and Higgins found a delay of conidial germination in *Colletrotichum coccodes* incubated with 0.1 mM SNP and an acceleration of germination with the addition of NO inhibitors [21]. NO gas at a low concentration also prevented growth of *Aspergillus niger*, *Monilinia fructicola,* and *Penicillium italicum* [22]. Hu et al. reported that NO directly inhibited the growth of *F. sulphureum* and cured and prevented dry rot of potato tubers [17]. These different results might be related to the different sensitivities of fungal species to NO.

Nevertheless, to date, there was limited information available about the effects of NO against postharvest disease and its related defense mechanisms in loquat fruit. The grey spot rot cause by *Pestalotiopsis eriobotryfolia* (Guba), determined by Chen et Chao, was one of the main diseases of loquat fruit, which was controlled mainly by the use of fungicides Sportak and Sporgon [23]. The objectives of this study were, firstly, to determine the influences of SNP on grey spot rot of postharvest loquat fruit caused by *P. eriobotryfolia*; secondly, to investigate the antifungal effects of SNP against *P. eriobotryfolia* in vitro test by assaying spore germination and mycelial growth. Furthermore, the effectiveness and defense response of SNP on antioxidant and defense-related enzymes, total phenolics, hydrogen peroxide (H_2_O_2_), cell wall components, and its hydrolase of loquat fruit inoculated with *P. eriobotryfolia* were tested in vivo. The results could provide a strategy for controlling loquat postharvest diseases and further understanding for the mechanisms behind the induced resistance in loquat fruit by SNP.

## 2. Results

### 2.1. Effects of SNP on Disease Incidence and Lesion Diameter of Loquat Fruit Inoculated with P. eriobotryfoli

**Figure 1:**
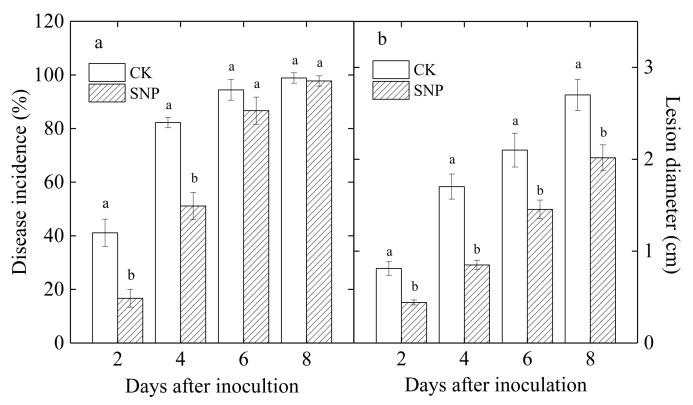
Disease incidence (**a**) and lesion diameter (**b**) of *P. eriobotryfolia* in loquat fruit during incubation at 20 °C. Data are presented as the mean ± SE of three replicates. The different letters at same time point indicate a significant difference between control and SNP treatment at *p* < 0.05.

After inoculation with *P. eriobotryfolia*, the disease incidence and lesion diameter increased gradually in SNP-treated and control loquat fruit (Figure 1). Although no significant difference was found in the disease incidence between control and SNP-treated fruits after inoculation for 8 d, SNP delayed the development of the disease caused by *P. eriobotryfolia* (Figure 1a). The disease incidence in SNP-treated loquat fruit was 41% and 62% of that in control on the second and fourth d of incubation, respectively (Figure 1a). SNP significantly reduced lesion diameter in loquat fruit inoculated with *P. eriobotryfolia* (Figure 1b). The lesion diameter on SNP-treated fruit was only 54, 50, 69, and 75% of that in control on the second, fourth, sixth, and eighth day of incubation, respectively (Figure 1b).

### 2.2. Effects of SNP on Mycelial Growth and Spore Germination of P. eriobotryfolia In Vitro

**Figure 2:**
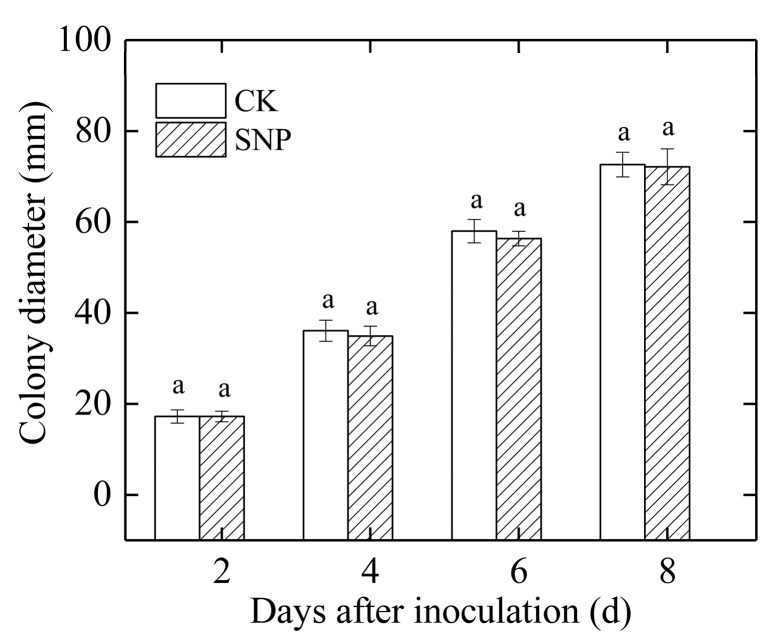
Effect of SNP on mycelial growth of *P. eriobotryfolia* in vitro incubated at 25 ± 1 °C. Data are presented as the mean ± SE of five replicates. The different letters at same time point indicate a significant difference between control and SNP treatment at *p* < 0.05.

The mycelial colony of *P. eriobotryfolia* of the control rapidly grew from 4 mm to 73 mm in diameter during 8 d of incubation. As compared with the control group, 50 μM SNP did not significantly affect the mycelial growth of *P. eriobotryfolia* (Figure 2). Similarly, the difference of spore germination and germ tube length of *P. eriobotryfolia* were not significant between SNP treatment and the control during 6 h and 12 h of incubation (Table 1).

### 2.3. Effect of SNP on Activities of SOD, CAT and APX as Well as H_2_O_2_ Content in Loquat Fruit

**Figure 3:**
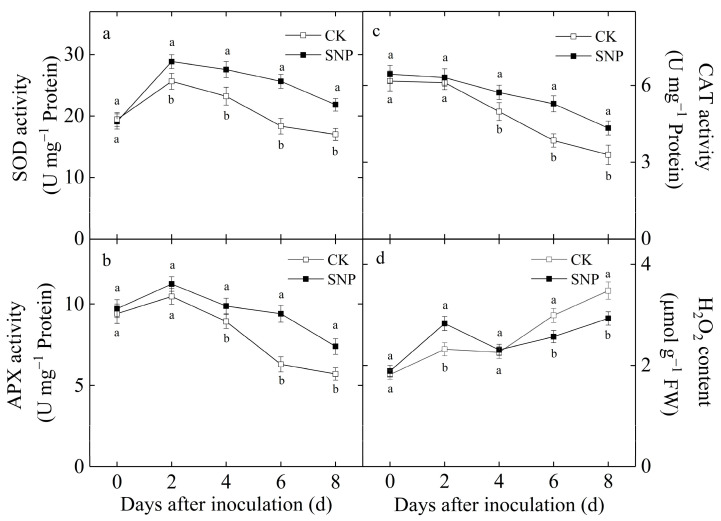
Effects of SNP treatment on the activities of SOD (**a**), APX (**b**), CAT (**c**) and H_2_O_2_ content (**d**) in loquat fruits during incubation at 20 °C. Data are presented as the mean ± SE of three replicates. The different letters at same time point indicate a significant difference between control and SNP treatment at *p* < 0.05.

As shown in Figure 3a,b, SOD, and APX activities in SNP-treated and control fruit increased firstly and then decreased dramatically with the incubation time extension and reached maximum values at two days after inoculation. SNP treatment enhanced the initial increase and inhibited the later decline in SOD and APX activities. At 8 d after inoculation, SOD and APX activities in SNP treated fruit were 28 and 30% higher than those of the control, respectively. CAT activity in SNP-treated and control fruit decreased gradually during the incubation with a significantly higher CAT activity in SNP-treated fruit after eight days of incubation, which was 28% higher on average than those in the control (Figure 3c). As shown in Figure 3d, SNP treatment significantly increased H_2_O_2_ content during the first 2 d of incubation and H_2_O_2_ content in SNP-treated fruit was 22% higher than that in the control. However, H_2_O_2_ content was significantly lower in SNP-treated fruit after 6 and 8 d of incubation, compared with the control, which was 87% and 84% of that in control.

### 2.4. Effects of SNP on the Activities of GLU, CHI, PAL, and PPO and Total Phenolic Content of Loquat Fruit Inoculated with P. eriobotryfolia

**Figure 4:**
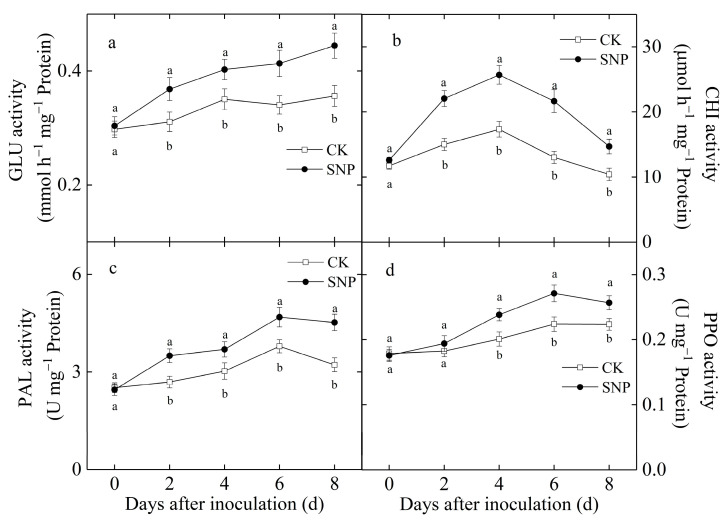
Effects of SNP treatment on GLU (**a**), CHI (**b**), PAL (**c**), and PPO (**d**) activities in loquat fruit during incubation at 20 °C. Data are presented as the mean ± SE of three replicates. The different letters at same time point indicate a significant difference between control and SNP treatment at *p* < 0.05.

GLU activity increased gradually in both control and SNP-treated fruit after inoculation with *P. eriobotryfolia* (Figure 4a). However, GLU activity in SNP-treated fruit was significantly higher than that in control fruit at the same time after inoculation. After 8 d of inoculation, SNP-treated fruit showed 1.25 times higher GLU activity than control fruit (Figure 4a).

CHI activity in the control fruit steadily continuously and reached the peak value on the fourth day of inoculation, followed by a sharp decline (Figure 4b). SNP-treated fruit with *P. eriobotryfolia* inoculation exhibited a similar trend of CHI activity to that in the control fruit, but the former had higher activity during inoculation (Figure 4b). CHI activity in SNP-treated fruit on the fourth day of incubation was 48% higher than that in the control (Figure 4b).

PAL activity in loquat fruit after inoculation with *P. eriobotryfolia* gradually increased, peaked after six days of inoculation, and then decreased slightly, where SNP-treated fruit displayed higher PAL activity during the incubation compared to the control (Figure 4c). The average PAL activity in SNP-treated fruit was 29% greater than that in the control group during incubation.

PPO activity in the control fruit rose steadily during the incubation. However, it reached the highest value in SNP-treated fruit on the sixth day; afterwards it decreased slightly (Figure 4d). In comparison to the control group, SNP treatment significantly enhanced the increase in PPO activity (*p* < 0.05) (Figure 4d).

**Figure 5:**
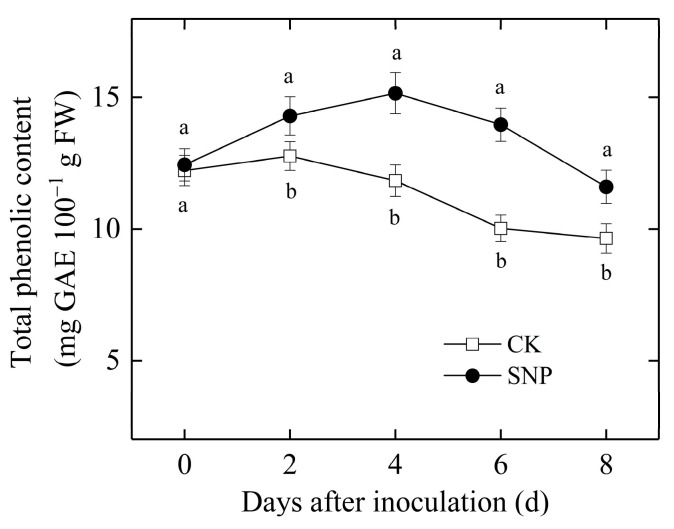
Effects of SNP treatment on total phenolics content in loquat fruit during incubation at 20 °C. Data are presented as the mean ± SE of three replicates. The different letters at same time point indicate a significant difference between control and SNP treatment at *p* < 0.05.

Total phenolics content in the control fruit increased initially with the increasing time of incubation, reached a peak value on the fourth day of inoculation, and then decreased markedly. It showed a similar pattern but was enhanced significantly and delayed the decline in SNP-treated fruit (Figure 5). The average activity in SNP-treated fruit was 25% higher than that of the control after inoculation.

### 2.5. Effects of SNP on PG, PME and Cellulase Activities of Loquat Fruit Inoculated with P. eriobotryfolia

**Figure 6:**
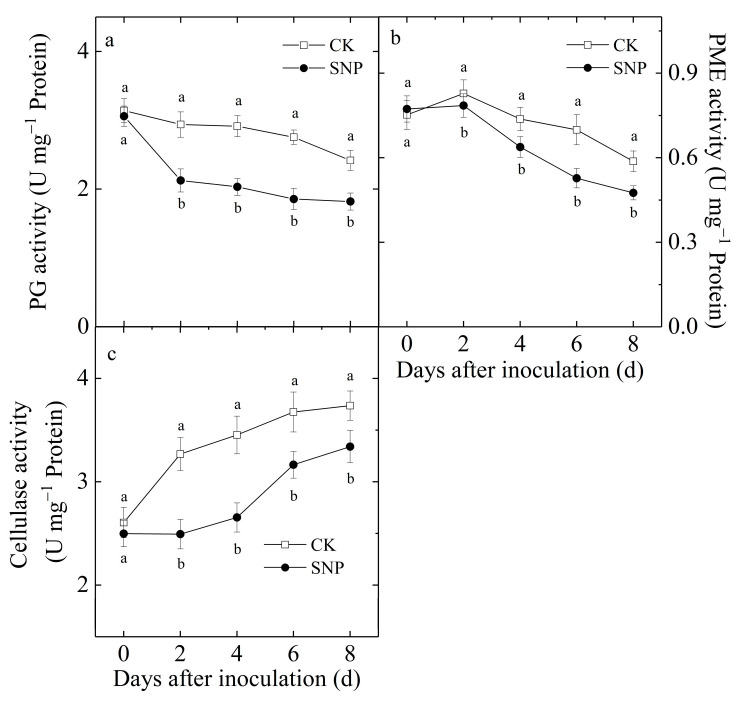
Effects of SNP on PG (**a**), PME (**b**), and cellulase (**c**) activity in loquat fruit during incubation at 20 °C. Data are presented as the mean ± SE of three replicates. The different letters at same time point indicate a significant difference between control and SNP treatment at *p* < 0.05.

PG activity in control loquat fruit declined slightly during incubation (Figure 6a). SNP caused a dramatic decline in PG activity through the whole incubation, especially at two days after inoculation. The average PG activity of SNP-treated fruit was 29% lower than that of the control fruit from two-to-eight days (Figure 6a).

PME activity in the control fruit increased slightly on the second day of inoculation, and then decreased gradually (Figure 6b). Compared with the control, SNP significantly reduced PME activity during incubation. The PME activity of SNP-treated fruit was 19% lower than that of control after 8 d of inoculation (Figure 6b).

Cellulase activity in both the control and SNP-treated fruit increased gradually during incubation (Figure 6c). Compared with the control, SNP significantly decreased the increase in cellulase activity, which was 11% lower in SNP-treated fruit than that in control fruit after 8 d of incubation.

### 2.6. Effects of SNP on Propectin, WSP and Cellulose Contents of Loquat Fruit Inoculated with P. eriobotryfolia

**Figure 7:**
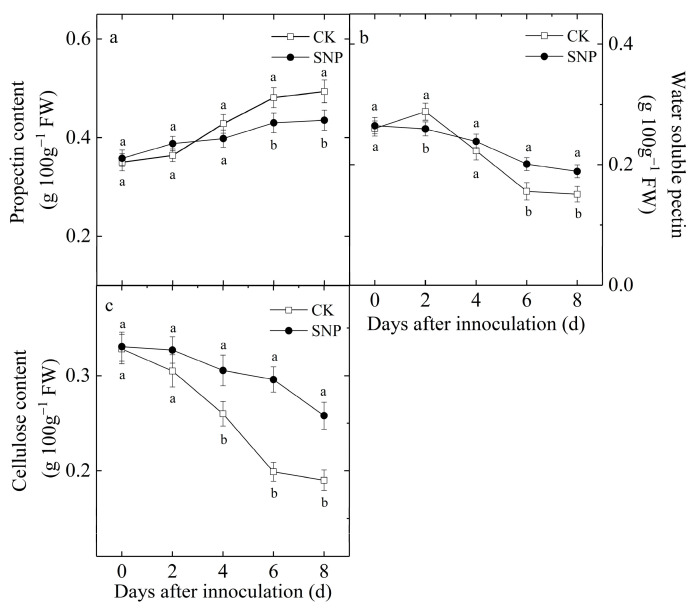
Effect of SNP on propectin (**a**), water-soluble pectin (**b**) and cellulose (**c**) content in loquat fruit during incubation at 20 °C. Data are presented as the mean ± SE of three replicates. The different letters at same time point indicate a significant difference between control and SNP treatment at *p* < 0.05.

Propectin content increased gradually in the control and SNP-treated fruit during incubation (Figure 7a), while the WSP content decreased gradually (Figure 7b). SNP treatment significantly inhibited the increase of propectin content and the decrease of WPS content after 4 d of incubation. At the end of the incubation, propectin content in the control fruit was 13% higher than that of SNP-treated fruit, while WPS content in the control fruit was 25% lower than that in SNP-treated fruit. Unlike propectin and WPS contents, cellulose contents in SNP-treated and control fruit decreased in similar patterns (Figure 7c). However, SNP treatment obviously delayed the decrease. Cellulose contents in SNP-treated and control fruit after 8 d of incubation decreased by 22% and 42%, respectively.

### 2.7. Principal Component and Pearson Correlation Analysis

**Figure 8:**
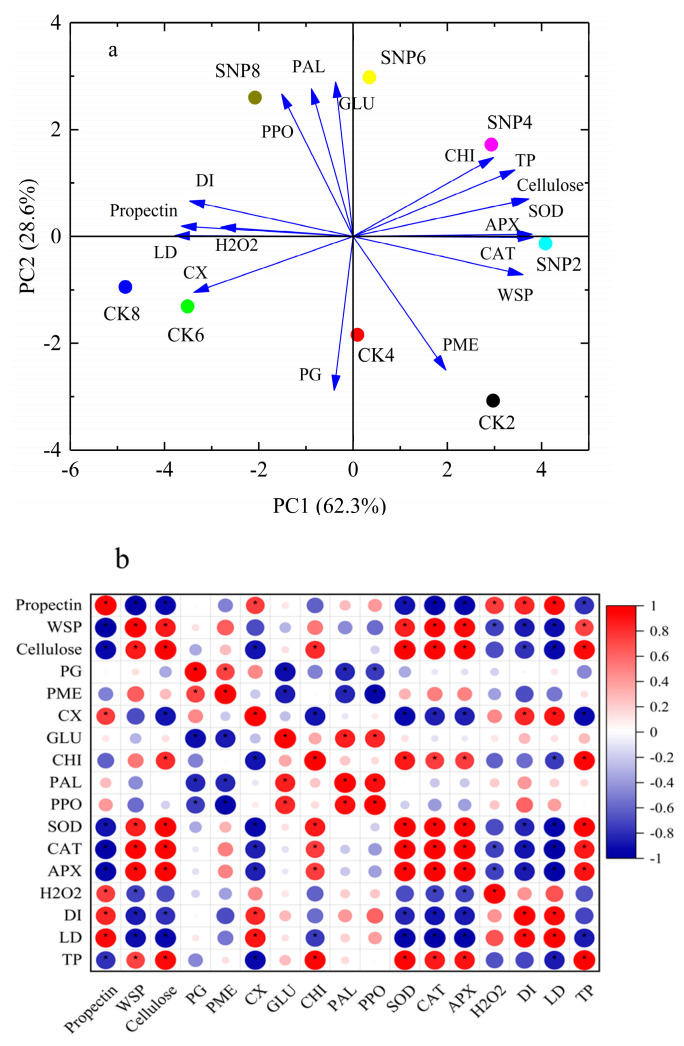
The bioplot for principal component analysis (**a**) and Pearson correlation analysis (**b**) of different response parameters in SNP-treated and control fruits after inoculation. Different abbreviations used in the figure are as follows: DI-disease incidence, LD-lesion diameter, TP-total phenolics. “*” means significant level at *p* < 0.05. Red and blue colors indicate strong positive and negative correlations, respectively.

The principal component analysis (PCA) was adopted to demonstrate the overall responses of fruit grey spot rot and physicochemical parameters to SNP treatment, compared with the control (Figure 8a). The PC1 and PC2 values of the PCA loading plot described 62.3 and 28.6% of total variance, respectively. PC1 tended to separate the effect of different times after inoculation, and PC2 segregated the effect of SNP from the control. To investigate the contributors to PC, the physiological loadings in PC1 and PC2 were compared. It was clear that PC1 was positively correlated with SOD, APX, CAT, TP, WSP, and CHI, and negatively correlated with LD, DI, CX, H_2_O_2,_ and propectin. PC2 was positively correlated with PAL, GLU, and PPO, and negatively correlated with PG and PME. Strong and positive correlations were found between the control and DI, LD, CX, PG, PME, and propectin. However, PCA revealed positive correlations between PPO, PAL, GLU, CHI, TP, SOD, CAT, APX, WSP, cellulose, and SNP treatment (Figure 8a). These results suggested that antioxidant enzymes, phenylpropanoid pathways, and pathogenesis-related proteins were critical in fighting against gray spot disease.

Pearson’s correlation analysis was used to investigate the relationship between disease incidence and physicochemical attributes in SNP-treated fruit (Figure 8b). It was observed that disease incidence (LD and DI) was significant positively correlated with propectin and CX, while significantly negatively correlated with WSP, cellulose, and antioxidant enzymes. Cellulose and WSP were strongly positively correlated with CHI, antioxidant enzymes, and TP. Propectin was strongly positively correlated with CX and H_2_O_2_. PAL was positively correlated with PPO and GLU. PME was positively correlated with PG. TP also showed positive correlations with WSP, cellulose, CHI, and antioxidant enzymes. However, propectin was strongly negatively correlated with WSP, cellulose, antioxidant enzymes, and TP. PME and PG were strongly negatively correlated with GLU, PAL, and PPO. CX was strongly negatively correlated with cellulose, CHI, and TP. WSP was also strongly negatively correlated with H_2_O_2_.

## 3. Discussion

NO is known to have a critical role in inducing disease resistance in several fruits [11,13,14,15,24,25]. In the present study, SNP treatment significantly reduced grey spot rot incidence (Figure 1a) and lesion diameter (Figure 1b) in loquat fruit inoculated with *P. eriobotryfolia*. Additionally, SNP had no significant inhibitory effect on mycelial growth (Figure 2) and spore germination of *P. eriobotryfolia* (Table 1). These results indicated that the inhibitory effect of SNP on *P. eriobotryfolia* in loquat fruit was a consequence of induced resistance. Our results were in agreement with the previous report, in which NO did not have direct inhibitory effect on growth of *Botrytis cinerea* in vitro, but was effective in controlling gray mold in tomato fruit [15]. Hu et al. also suggested that exogenous NO did not inhibit mycelial growth and spore germination of *C. gloeosporioides* in vitro, but it could reduce the anthracnose incidence and severity in mango fruit [19]. Similarly, Gu et al. found that 15 μM of NO did not significantly inhibit spore germination and germ tube length of *Monilinia fructicola,* but significantly reduced disease incidence and lesion areas in peach fruit [20].

The protection of fruit from invasion of fungal pathogens is largely due to activation of a highly coordinated biochemical and structural defense system that helps ward off the spread of pathogens [26]. H_2_O_2_ accumulation has the potential to exert various effects on plant defense responses [26]. SOD, CAT, and APX are significant enzymes in regulating H_2_O_2_ generation. It has been reported that the resistance to anthracnose rot of loquat fruit treated with methyl jasmonate (MeJA) was related to the accumulation of H_2_O_2_ during early infection [26]. Salicylic acid (SA) treatment increased the H_2_O_2_ content which was mediated by an inhibition of CAT and APX activities, and enhanced the resistance to anthracnose rot of mango fruit [8]. However, excess H_2_O_2_ have been implicated in the destruction of plant cells through peroxidation of lipids [10,27]. Our results also showed that SNP treatment remarkably induced the increase in SOD activity in early incubation whereas inhibited the decline in CAT and APX activity in later incubation (Figure 3), contributing to an enhanced level of H_2_O_2_ in loquat fruit (Figure 3). These results suggested that the increase in H_2_O_2_ content as a signaling molecule for the induction of disease resistance on loquat fruit in the early period of incubation and the reduction in oxidative damage in the later period of incubation might be one of the mechanisms that enhanced pathogen resistance in SNP-treated loquat fruit.

PAL is an essential enzyme in the phenylpropanoid biosynthesis pathway leading to the synthesis of lignins, phytoalexins, phenols, etc. [16]. PPO participates in the lignification of plant cells and the oxidation of polyphenols into quinines with strong antifungal properties during a microbial invasion [4]. In addition, phenolic compounds can restrict the pathogen growth [25]. The present results study showed SNP increased the activities of PAL (Figure 4c) and PPO (Figure 4d), as well as total phenolics content (Figure 5) in loquat fruit inoculated with *P. eriobotryfolia* compared with the control, while being accompanied with a lower disease incidence (Figure 1a) and lesion diameter (Figure 1b). An increase in the activities of these enzymes and phenolic content was also observed in loquat fruit treated by ethanol and ultrasonic combined with peracetic acid [4,6]. The accumulation of total phenolics by NO treatment was also observed in pitaya fruit [13], kiwifruit [11], and citrus fruit [14], resulting in increased disease resistance.

PR protein, particularly CHI and GLU, are thought to provide defense against fungal pathogens by degrading a pathogen’s cell wall [27]. In the current study, SNP caused an evident raise in GLU and CHI activities in loquat fruit (Figure 4a,b), which implied that CHI and GLU might play important roles in decomposing the fungal cell wall of *P. eriobotryfolia*, consequently inhibiting disease development in loquat fruit (Figure 1a). In line with our results, enhanced resistance against postharvest anthracnose in relation to higher CHI and GLU activities were also observed in ethanol-treated loquat fruit inoculated with *C. acutatum* during storage [4]. The increase in GLU and CHI activities and the reduction in disease severity by SNP treatment were also observed in peaches [20], tomatoes [24], kiwifruits [11], and pitayas [13]. Therefore, we deduced that the enhanced activities of defense enzymes could be one of the mechanisms of SNP in inducing disease resistance against grey spot rot in loquat fruit.

The plant cell wall represents the first barrier opposed to pathogens. The solubilization and depolymerization of cell wall constituents would facilitate the infections by postharvest pathogens, increase postharvest decay and decrease the quality of fruit [28]. Therefore, the cell wall integrity affects plant susceptibility to pathogens. Pectin and cellulose are main cell wall of constituents, which are important determinants of cell wall thickness and glue cells together [29]. He et al. reported that SA treatment effectively reduced disease incidence in mango fruit during storage, which might be associated with suppressing degradation of pectin [8]. In addition, hot water treatment effectively maintained fruit firmness of muskmelon by suppressing the activities of endo-1,4-D-glucanase (EGase), PG, and PME, which contributed to the reduced decay incidence [29]. Sinha et al. also found that relatively lower cell wall hydrolase activities in pear during storage under salicylic acid enriched beeswax coatings treatment contributed to enhance disease resistant ability [30]. The present study showed that SNP increased the propectin and cellulose contents (Figure 7a,c) and inhibited the decrease of water-soluble pectin (Figure 7b) in loquat fruit during storage after inoculation with *P. eriobotryfolia*, which was attributed to the inhibited effect of SNP treatment on the activities of PG, PME, and cellulase (Figure 6). These results suggested that SNP could reduce the degradation of pectin and cellulose through inhibiting the cell wall hydrolase activities and consequently prevent the pathogen from spreading. Previous studies have also shown that NO decreased PME and PG activities in papaya [31] and cherry fruit [32].

PCA is a powerful approach for multivariate studies, and it has been used to evaluate the influence of SNP on disease incidence and physico-biochemical indexes [33]. In the present research, the PCA results showed a distinct separation among disease incidence and physico-biochemical parameters on PC1 and PC2, respectively (Figure 8a). Furthermore, the discrete plots between the control and SNP on PC2 indicated that the main influence of SNP on loquat gray spot disease rot was phenylpropanoid pathway, pathogenesis-related proteins, and pectin hydrolysis (Figure 8b). Pearson correlation depicted strong negative correlation of disease incidence with antioxidant enzymes, pectin hydrolysis, and total phenolics.

## 4. Materials and Methods

### 4.1. Fruit Materials

Loquat (*E. japonica* L. cv. Wuxing) fruits were hand-harvested at the mature stage from an orchard in Kaiyang, Guizhou Province, China. Fruit used for experiment were selected for uniform size and the absence of physical injuries or infections.

### 4.2. Pathogen

*P. eriobotryfolia* was isolated from naturally infected loquat fruit showing typical gray spot lesions through culture and re-culture of single macroconidia on potato dextrose agar (PDA). After identifying morphologically and pathogenically, the single spore culture was maintained on PDA medium in the dark at 4 °C. Spores of *P. eriobotryfolia* were obtained by flooding the 2-week-old cultures incubated at 25 ± 1 °C with sterile distilled water. The concentration of spore suspensions was adjusted to 2 × 10^6^ spores/mL with a hemocytometer.

### 4.3. SNP Treatment and Inoculation

In our previous experiment, 50 μM of SNP (NO donors, Sigma-Aldrich Trading Co., Ltd., Shanghai, China) significantly delayed the senescence and quality deterioration in “Wuxing” loquat fruit during storage [34]. Therefore, a SNP concentration at 50 μM was employed in this experiment. Selected loquat fruit were disinfected with 0.05% sodium hypochlorite solution for 5 min, rinsed with distilled water, air-dried, and then randomly divided into two groups. Loquat fruit were immersed in 0 and 50 μM SNP aqueous solutions for 10 min [34]. After 24 h, loquat fruit were wounded (3 mm deep × 4 mm wide) using a sterile nail at the equator of each fruit. Then, 20 μL of spore suspension at the concentration of 2 × 10^6^ spores/mL were inoculated into each wound. After air-drying for 1 h at room temperature, the inoculated fruit were placed into plastic boxes and stored at 20 °C and 90–95% humidity for 8 d. Each treatment contained three replicates with 30 loquat fruit per replicate. The entire experiment was conducted twice. Disease incidence and lesion diameter were measured at 2, 4, 6, and 8 d after inoculation. When the visible rot zone beyond the wound area on each fruit was more than 2 mm wide, it was considered as an infected fruit [4]. Another batch of loquat fruit with the same treatment and inoculated with *P. eriobotryfolia* was stored at same condition to investigate defense-related and antioxidant enzymes, total phenolics, cell wall components, and cell wall-related enzymes. The pulp samples from each treatment were collected at 5–15 mm from the edge of the inoculated lesion of the loquat fruit at various time intervals (0, 2, 4, 6, and 8 d after inoculation). Tissue samples were mixed and frozen immediately in liquid nitrogen and stored at −78 °C until use. Each treatment was performed in three replicates with 75 fruit per replicate.

### 4.4. SOD, CAT and APX Activities and H_2_O_2_ Content

All enzyme extract procedures were conducted at 4 °C. One gram of sample was homogenized in 5 mL ice-cold 50 mM sodium phosphate buffer (pH 7.0) containing 1.33 mM EDTA and 1% (*w/v*) polyvinyl pyrrolidone. The homogenate was then centrifuged at 12,000× *g* for 20 min, and the supernatant was used for enzyme analysis. The activities of SOD, CAT, and APX were assayed by the method of Ren et al. [25]. One unit (U) of SOD activity was defined as the amount of enzyme that caused a 50% inhibition of nitroblue tetrazolium (NBT) reduction. One unit (U) of CAT and APX was defined as the change in 0.01 and 0.1 absorbance units per minute at 240 nm and 290 nm with a spectrophotometer, respectively. The unit of SOD, CAT, and APX activity was expressed as U mg^−1^ protein. The protein content in the supernatant was measured according to the Bradford method.

To measure H_2_O_2_ content, 1 g of sample was homogenized with 5 mL of chilled acetone and then centrifuged at 12,000× *g* for 20 min at 4 °C. The supernatant was collected immediately for H_2_O_2_ analysis. The content of H_2_O_2_ was determined according to the method of He et al. by monitoring the absorbance of the titanium-peroxide complex at 410 nm [9].

### 4.5. Total Phenolics Content

Total phenolics content of pulp tissue was determined spectrophotometrically using Folin–Ciocalteu reagent following the method of He et al. and expressed as l mg gallic acid equivalent 100 g^−1^ FW [8].

### 4.6. Pectin and Cellulose Content

Protopectin, water soluble pectin (WSP), and cellulose contents of pulp tissue were extracted and determined as described by He et al. [8].

### 4.7. Cell Wall-Related Enzymes

Polygalacturonase (PG), cellulase, and pectin methylesterase activities of pulp tissue were assayed according to the previous method [4]. One U of PG and cellulase activity was defined as the amount of enzyme that liberated 1 mg of galacturonic acid and glucose for one hour per g FW, respectively. One U of PME activity was calculated as 1 mM NaOH consumed for one hour per mg protein.

### 4.8. Disease Resistance-Related Enzyme Activities

Phenylalanine ammonialyase (PAL) and polyphenol oxidase (PPO) activities were determined according to Song et al. [27]. One U of PAL and PPO activities was spectrophotometrically determined and expressed as 0.01 and 1.0 increases in absorbance at 290 nm per hour and 420 nm per min, respectively.

Chitinase (CHI) and β-1,3-Glucanase (GLU) activities were determined according to the method of Ren et al. [25]. One U of CHI and GLU activities was defined as mmol N-acetyl-D-glucosamine (NAG) and glucose h^−1^ mg^−1^ protein, respectively.

### 4.9. Effect of SNP on Mycelial Growth and Spore Germination of P. eriobotryfolia In Vitro

#### 4.9.1. Mycelial Growth

The effect of SNP on mycelial growth of *P. eriobotryfolia* was studied on PDA in Petri dishes according to the method of He et al. [8]. Mycelial growth as expressed by diameter (mm) was recorded at 2, 4, 6, and 8 days of incubation at 25 ± 1 °C. Each treatment contained five replicates, and the experiment was repeated twice.

#### 4.9.2. Spore Germination and Germ Tube Elongation

The effects of SNP on spore germination and germ tube length of the pathogen were investigated in potato dextrose broth (PDB) using the method of Ren et al. [25]. About 150 spores per replicate were observed microscopically to determine germination rate and germ tube length at 6 and 12 h after incubation. Spores were considered germinated when germ tube length was equal to or greater than spore length. All treatments consisted of three replicates, and the experiment was repeated twice.

### 4.10. Statistical Analysis

The experiments were performed using a completely randomized design. Data were expressed as mean ± SD from a typical single experiment and analyzed by one-way analyses of variance (ANOVA) with the SPSS 16.0 software (SPSS Inc., Chicago, IL, USA). Differences at *p* < 0.05 were statistically significant. Principal component analysis (PCA) and Pearson’s correlation analysis were performed using origin 2021.

## 5. Conclusions

SNP was effective in control of grey spot rot caused by *P. eriobotryfolia* in loquat fruit by altering H_2_O_2_ metabolism, enhancing pathogenesis-related enzymes activity, and accumulating total phenolics, mediating cell wall metabolism. However, SNP had no direct inhibition of pathogen growth of *P. eriobotryfolia* in vitro. The study suggested that an exogenous application of NO might be recommended as an effective strategy to control postharvest disease of loquat fruit.

## Figures and Tables

**Table 1:** Effects of SNP on spore germination and germ tube elongation of *P. eriobotryfolia* in medium.

SNP (μM)	6 h		12 h	
	Spore Germination (%)	Germ Tube Length (μm)	Spore Germination (%)	Germ Tube Length (μm)
0 (CK)	23.9 ± 2.9 a	48.6 ± 7.6 a	90.3 ± 4.1 a	73.9 ± 17.2 a
50	20.8 ± 2.7 a	44.0 ± 9.3 a	86.1 ± 3.9 a	65.8 ± 14.5 a

Note: Data followed by the same letters at same time point are not significantly different between control and SNP treatment at *p* > 0.05.

## Data Availability

All relevant data supporting the findings of this study are included in this article. Correspondence and requests for materials should be addressed to Y.R. and J.H.
